# Parallel Evolution towards Increased Motility in Long-Term Cultures of Escherichia coli, Even Though Motility was Not Required for Long-Term Survival

**DOI:** 10.1128/spectrum.02330-21

**Published:** 2022-06-23

**Authors:** Autumn L. Henderson, Angie Moreno, Karin E. Kram

**Affiliations:** a Department of Biology, California State University, Dominguez Hills, Carson, California, USA; South China Sea Institute of Oceanology

**Keywords:** motility, evolution, RNA-seq, transcriptome, bacterial genetics

## Abstract

Escherichia coli can survive for long periods in batch culture in the laboratory, where they experience a stressful and heterogeneous environment. During this incubation, E. coli acquires mutations that are selected in response to this environment, ultimately leading to evolved populations that are better adapted to these complex conditions, which can lead to a better understanding of evolutionary mechanisms. Mutations in regulatory genes often play a role in adapting to heterogeneous environments. To identify such mutations, we examined transcriptional differences during log phase growth in unaged cells compared to those that had been aged for 10 days and regrown. We identified expression changes in genes involved in motility and chemotaxis after adaptation to long-term cultures. We hypothesized that aged populations would also have phenotypic changes in motility and that motility may play a role in survival and adaptation to long-term cultures. While aged populations did show an increase in motility, this increase was not essential for survival in long-term cultures. We identified mutations in the regulatory gene *sspA* and other genes that may contribute to the observed differences in motility. Taken together, these data provide an overall picture of the role of mutations in regulatory genes for adaptation while underscoring that all changes that occur during evolution in stressful environments are not necessarily adaptive.

**IMPORTANCE** Understanding how bacteria adapt in long-term cultures aids in both better treatment options for bacterial infections and gives insight into the mechanisms involved in bacterial evolution. In the past, it has been difficult to study these organisms in their natural environments. By using experimental evolution in heterogeneous and stressful laboratory conditions, we can more closely mimic natural environments and examine evolutionary mechanisms. One way to observe these mechanisms is to look at transcriptomic and genomic data from cells adapted to these complex conditions. Here, we found that although aged cells increase motility, this increase is not essential for survival in these conditions. These data emphasize that not all changes that occur due to evolutionary processes are adaptive, but these observations could still lead to hypotheses about the causative mutations. The information gained here allow us to make inferences about general mechanisms underlying phenotypic changes due to evolution.

## INTRODUCTION

In the natural environment, organisms regularly encounter heterogeneous and stressful environments. Studying how a species evolves can provide insight into why species have certain traits and help explain different adaptive strategies that lead to survival and increased fitness in stressful environments (1). Changes that occur during growth in new environments can either be sustained, increased, or removed by natural selection or genetic drift (1). Changes can also be driven by correlated evolution, where two or more traits are driven together by the evolutionary process (2).

Escherichia coli is an ideal model organism for studying evolution and adaptation, not only because it grows rapidly but also because it is quite versatile and genetically diverse (3). Because there are infectious strains of E. coli, we must understand how it evolves in different environments to produce more effective antibiotics. Studying E. coli is beneficial for not only the development of more targeted treatments for infection but also for insight into basic biology, such as adaptive mechanisms.

E. coli, like many microbial organisms, has a five-phase life cycle in the laboratory: lag phase, log or exponential-phase, stationary-phase, death phase, and long-term stationary-phase (LTSP) (4). LTSP is a complex environment, where the total number of cells remains relatively constant because the replication and death rates of cells in this phase are balanced. During LTSP, cells undergo multiple stresses such as starvation, oxidative stress, and changes in pH (5). During this phase, different populations that have acclimated or adapted to the environment grow while other populations died, creating new conditions that allow new populations to grow (4). The continuous cycling of nutrients and waste products creates a heterogeneous, complex, and stressful environment in which cells need to adapt to survive. As the E. coli population ages, mutations that allow for better survival in stressful environments such as LTSP are selected for and can increase in frequency (4). While we and others have begun to explore this environment, the exact changes and stresses cells experience during LTSP are mostly unknown (6–10).

Cells can respond to their environment by altering gene expression in an impermanent way (physiological acclimation), but they can also adapt by accumulating mutations that permanently change expression for one or multiple genes. Mutations that affect gene expression have frequently been identified as one of the actors of evolutionary change, indicating that mutations in *trans* regulatory factors may play a role in adaptive evolution in multiple organisms and environments (11–13). One way to explore the conditions cells experience during this stressful period and determine what regulatory factors may help cells adapt to these stresses, is to examine transcriptional changes that occur after cells have been incubated into LTSP.

To identify transcriptional differences between unaged E. coli and cells aged into LTSP, we performed RNA-seq (14) on log-phase cells of both aged and unaged populations. Because we previously identified regulatory changes in LTSP (15), we specifically aimed to identify transcriptional changes caused by mutations in the genome accumulating during long-term incubation. Comparing aged and unaged populations when grown into log-phase allowed us to differentiate permanent transcriptional changes caused by mutations in the genome and those caused by plastic changes due to normal regulatory systems in the cell. We identified multiple genes involved in motility and chemotaxis that were upregulated in cells that had adapted to LTSP. Based on these data, we hypothesized that motility would be increased in cells that were adapted to LTSP, and that motility would be important for cells to survive LTSP. Our data supported our first hypothesis that aged cells were more motile but not the second because motility was not essential for survival in LTSP. We also identified mutations that may cause changes in motility and other expression differences. These data help to understand the types of changes that occur during survival in heterogeneous and stressful environments.

## RESULTS

### Multiple genes were differentially expressed in aged populations, including many involved in motility.

To determine if incubation into LTSP led to differences in gene expression, we compared whole transcriptomes of log-phase cells (4 h) that were previously aged for 10 days (aged populations) to their parent strain which had not been previously aged (unaged populations). To ensure that these strains were in the same phase, we compared timing in lag, log, and entrance to stationary-phase and found no differences (Fig. S1). We identified 64 total genes that were differentially expressed (absolute log_2_ change of at least 2 and q-value < 0.05) between the two populations (Table 1). Thirty-five of these genes had higher expression in aged populations, whereas 29 genes had higher expression in unaged populations. Fourteen of the genes more expressed in unaged cells were related to resistance to acid stress (out of 38 genes categorized with the response to elevated pH GO terms in E. coli), and 27 of the more expressed genes in aged cells were related to motility and chemotaxis (out of 50 genes involved in these processes).

**TABLE 1:** Differentially expressed genes between unaged and aged populations of E. coli

Gene	Log_2_ fold change aged/unaged	q-value	Description
Upregulated in aged cells			
Genes involved in chemotaxis and motility			
*flgB*	4.0	1.4 × 10^−9^	flagellar basal-body rod protein
*flgC*	3.9	6.5 ×10^−10^	flagellar basal-body rod protein
*fliC*	3.7	3.3 × 10^−15^	flagellar filament structural protein
*flgE*	3.5	2.0 × 10^−15^	flagellar hook protein
*flgF*	3.5	1.8 × 10^−10^	flagellar basal-body rod protein
*tar*	3.5	1.6 × 10^−9^	methyl-accepting chemotaxis protein
*flgD*	3.4	1.3 × 10^−12^	flagellar biosynthesis, initiation of the hook assembly
*tap*	3.3	1.0 × 10^−9^	methyl-accepting chemotaxis protein - dipeptide-sensing
*flgG*	3.2	1.2 × 10^−11^	flagellar basal-body rod protein
*pdhE*	3.1	6.4 × 10^−6^	c-di-GMP phosphodiesterase involved in the regulation of the switch from flagellar motility to sessile behavior
*cheA*	3.0	4.1 × 10^−11^	chemotactic and aerotactic two-component signal transduction system histidine protein kinase
*fliF*	2.9	7.3 × 10^−6^	flagellar basal-body MS-ring and collar protein
*fliN*	2.8	4.5 × 10^−4^	flagellar motor switch protein
*fliD*	2.7	1.6 × 10^−6^	flagellar filament capping protein
*fliA*	2.7	4.2 × 10^−5^	sigma 28 (sigma F) factor responsible for genes involved in motility and flagellar synthesis
*flgH*	2.6	6.1 × 10^−6^	flagellar L-ring protein
*cheW*	2.4	6.0 × 10^−7^	chemotactic and aerotactic two-component signal transduction system coupling protein
*fliG*	2.3	7.2 × 10^−5^	flagellar motor switch protein
*fliH*	2.3	1.1 × 10^−2^	flagellar biosynthesis protein
*cheB*	2.3	1.8 × 10^−4^	chemotactic and aerotactic two-component signal transduction system response regulator
*flgK*	2.3	1.7 × 10^−5^	flagellar hook-filament junction protein 1
*fliS*	2.1	1.1 × 10^−2^	flagellar biosynthesis protein
*motB*	2.1	1.6 × 10^−3^	motility protein B
*flgI*	2.0	3.6 × 10^−3^	flagellar P-ring protein
*fliL*	2.0	9.0 × 10^−3^	flagellar protein
*ycgR*	2.0	1.3 × 10^−3^	flagellar brake protein
*flgL*	2.0	3.4 × 10^−5^	flagellar hook-filament junction protein 2
Genes involved in transport			
*ompF*	3.1	7.6 × 10^−14^	outer membrane porin F
*nupG*	2.8	1.6 × 10^−14^	nucleoside:H+ symporter
*tsx*	2.5	3.5 × 10^−10^	nucleoside-specific channel-forming protein
*gntP*	2.3	1.8 × 10^−8^	fructuronate transporter
Genes involved in nucleoside metabolism			
*cdd*	2.7	3.2 × 10^−5^	cytidine/deoxycytidine deaminase
*udp*	2.5	1.7 × 10^−8^	uridine phosphorylase
Unknown function			
*ydiL*	2.8	5.0 × 10^−9^	unknown
*ycdZ*	2.1	7.3 × 10^−3^	unknown
Downregulated in aged cells			
Genes involved in acid resistance			
*gadB*	−3.8	5.8 × 10^−19^	glutamate decarboxylase B
*dctR*	−3.7	8.4 × 10^−11^	putative DNA-binding transcriptional regulator
*gadC*	−3.7	1.3 × 10^−15^	L-glutamate:4-aminobutyrate antiporter
*yhiM*	−3.6	3.7 × 10^−15^	unknown
*gadA*	−3.4	1.6 × 10^−14^	glutamate decarboxylase A
*hdeD*	−3.3	7.8 × 10^−9^	acid-resistance membrane protein
*gadY*	−3.2	2.6 × 10^−9^	small regulatory RNA
*hdeB*	−3.1	8.4 × 10^−11^	periplasmic acid stress chaperone
*gadE*	−3.1	4.1 × 10^−12^	DNA-binding transcriptional activator
*hdeA*	−3.0	1.3 × 10^−9^	periplasmic acid stress chaperone
*slp*	−3.0	1.2 × 10^−13^	starvation lipoprotein
*gadX*	−3.0	1.2 × 10^−10^	DNA-binding transcriptional dual regulator
*gadW*	−2.6	5.9 × 10^−10^	DNA-binding transcriptional dual regulator
*glsA*	−2.1	2.1 × 10^−8^	glutaminase 1
Genes involved in other stress responses			
*yhiD*	−4.4	1.3 × 10^−15^	unknown
*ybaT*	−2.3	3.1 × 10^−8^	putative transporter
*aidB*	−2.2	1.7 × 10^−6^	isovaleryl-CoA dehydrogenase and DNA-binding transcriptional repressor
*cbpA*	−2.0	3.5 × 10^−7^	curved DNA-binding protein
*appY*	−2.0	5.1 × 10^−4^	DNA-binding transcriptional activator
Genes involved in metabolism			
*hyaA*	−2.3	1.7 × 10^−5^	hydrogenase 1 small subunit
*hyaF*	−2.1	2.1 × 10^−8^	unknown
*sufC*	−2.1	9.2 × 10^−5^	Fe-S cluster scaffold complex subunit
Genes involved in transport			
*mdtE*	−3.1	3.2 × 10^−6^	multidrug efflux pump membrane fusion protein
*mdtF*	−2.9	5.4 × 10^−6^	multidrug efflux pump RND permease
*lsrC*	−2.2	4.9 × 10^−5^	autoinducer-2 ABC transporter membrane subunit
Genes involved in motility			
*csgD*	−2.0	9.7 × 10^−5^	DNA-binding transcriptional dual regulator
Genes involved in other functions			
*lysQ*	−2.8	1.5 × 10^−2^	tRNA-Lys(UUU)
*cnu*	−2.7	6.6 × 10^−7^	H-NS- and StpA-binding protein
*ryeA*	−2.2	6.9 × 10^−3^	small antisense RNA

One of the motility-related genes, *fliA*, encodes a sigma factor (sigma 28/sigma F) that is responsible for the initiation of transcription of many genes involved in motility and flagellar synthesis (16). *fliA* was expressed 6.7 times more highly in aged populations than in unaged populations and was responsible for the transcription of 19 out of the 27 motility-related genes that were upregulated in aged populations (Table 1); (17). Further, *csgD*, which was expressed at 4-fold lower levels in aged populations, encodes a repressor of *fliA* (18).

Other categories of genes that had greater expression in aged populations included multiple genes encoding proteins involved in nucleoside transport or metabolism (*nupG*, *tsx*, *cdd*, and *udp*) (19), as well as other transport proteins (*ompF* and *gntP*) (20, 21). Genes that were expressed at a lower level in aged populations included those encoding proteins involved in stress responses, anaerobic respiration (*hyaA* and *hyaF* 22); the efflux pump MdtEF (23), and a quorum-sensing transporter LsrC (24).

### Cells aged to long-term stationary-phase were more motile than unaged cells.

**FIG 1:**
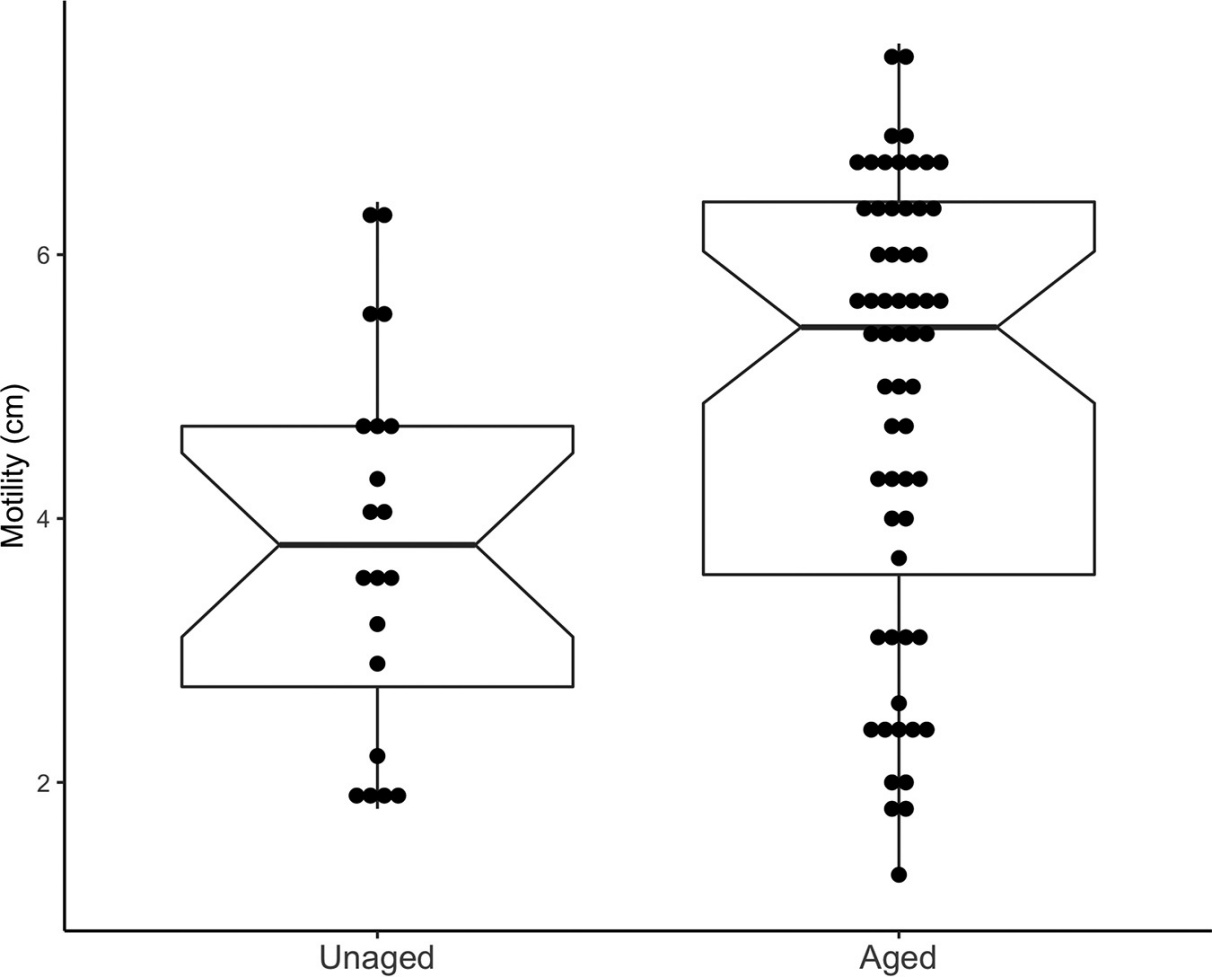
Motility is increased in aged populations of cells. Motility assays were performed on cells that were first aged for 10 days into LTSP as well as the parental strain of those populations that were unaged. Motility was measured in centimeters (cm) for 20 replicates of unaged populations and 60 replicates of aged populations. A Welch’s *t* test showed a significant difference between the groups: t(38) = −2.7, *P* = 0.01.

We hypothesized that the higher expression of motility-related genes described above would lead to more motile cells phenotypically. To test this hypothesis, we performed motility assays on both unaged and aged populations. To have more biological replicates and determine if this phenomenon would be re-evolved in independent cultures, we aged 6 new cultures for 10 days into LTSP (see Materials and Methods). We then saved these strains and regrew them for motility assays so that any phenotypic change observed would be due to genotypic changes from incubation into LTSP, not physiological acclimation. The average zone of motility for the unaged populations (*n* = 20) strain was 3.8 cm and it was 4.9 cm for the new aged strains (*n* = 60) (Fig. 1), which represents a significant increase in motility of 29% (Welch’s *t* test; t[38] = −2.7; *P* = 0.01) and recapitulation of the increased motility phenotype.

### Motility was not necessary for survival in LTSP.

**FIG 2:**
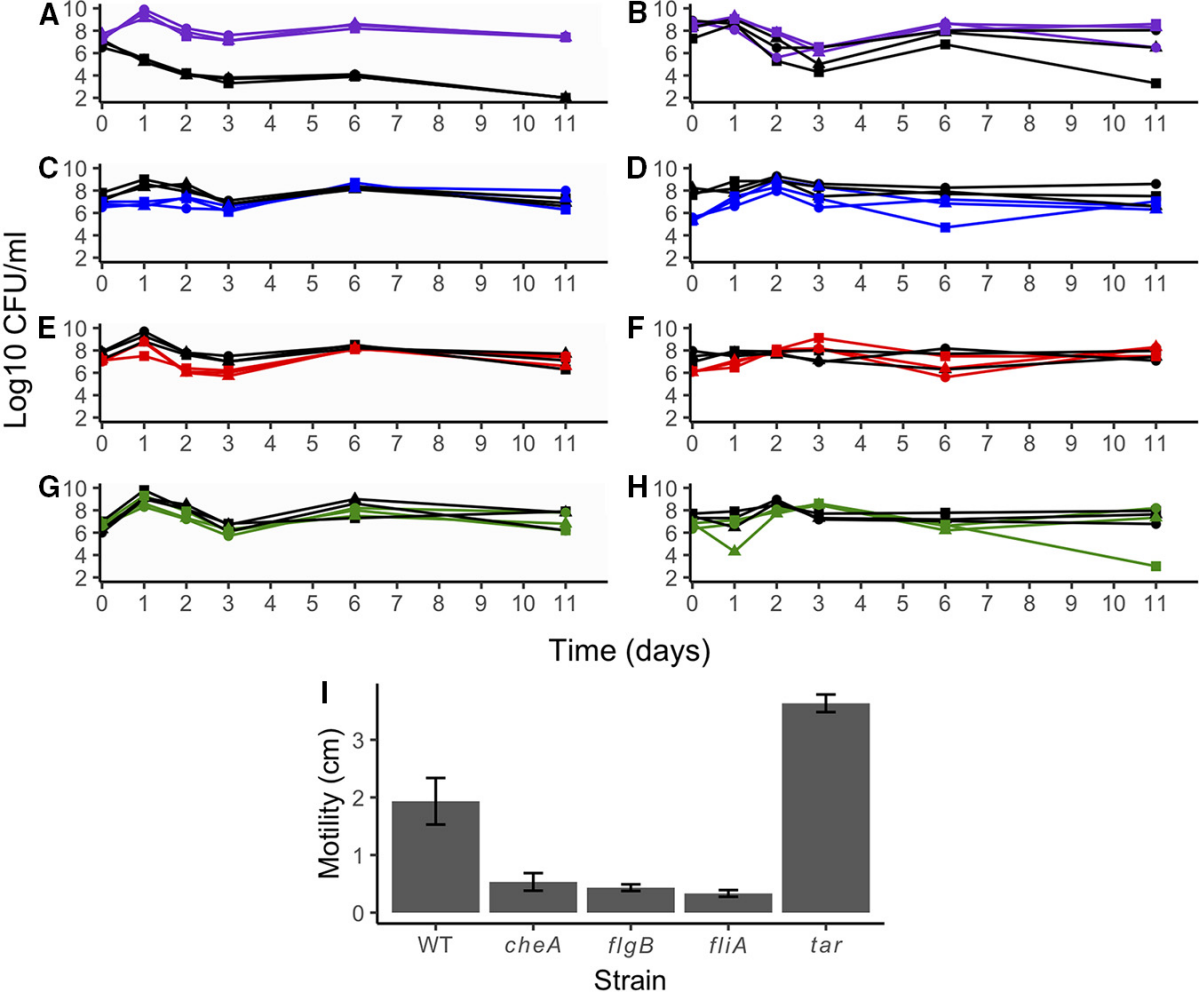
Motility is not correlated with survival or advantage in LTSP. (A to H) Wild type (WT) cells (black lines) competed with mutant strains (Δ*cheA*, purple; Δ*flgB*, blue; Δ*fliA*, red; Δ*tar*, green) by inoculating cultures in a 1:1 ratio and incubating them for 10 days in LTSP. Cultures were either coinoculated in a 1:1000 ratio with fresh LB after overnight growth (A, C, E, and G), and, therefore, competed during outgrowth or after 4 days of incubation in monoculture (B, D, F, and H) and only during LTSP. If the loss of the gene in the mutant strain did not affect survival in LTSP, we would have expected to see the WT strain and mutant strain at similar levels throughout the growth curve. Symbols represent the same culture for each of the three competitions. (I) Motility assays were performed with the WT strain and each mutant strain with the zone of motility measured at 12 h. The bar represents the average of three replicates of each strain, and the error bars represent the standard deviation. Each mutant strain’s motility measurement was significantly different than the wild-type strain (Student's *t* test; *P* < 0.005).

We hypothesized based on the transcriptomic and motility data that the increase in motility may be advantageous to cells in LTSP. To test this hypothesis, we chose four of the genes upregulated in long-term cultures which contribute to motility (*cheA*, *flgB*, *fliA*, and *tar*) (17), and competed strains missing each of these genes with wild-type cultures. These four genes were each in different operons and code for proteins that were in different parts of the flagellar biosynthesis or chemotaxis pathways. To confirm that the loss of these genes affected motility in our background strain in a way we expected, we first confirmed that mutant strain cell densities were similar to the cell density seen in WT cultures during lag, log, and stationary-phase (Fig. S1) and then performed motility assays for these strains (Δ*cheA*, Δ*flgB*, Δ*fliA*, and Δ*tar*) (Fig. 2I). The *ΔfliA* and *ΔflgB* mutants showed significantly less movement than the wild-type after 12 h, which was expected as neither of these strains should be able to form flagella. The *ΔcheA* mutant also had significantly less movement than the wild-type strain after 12 h (Fig. 2I). However, the *Δtar* mutant had a zone of motility of 3.6 cm after 12 h, significantly more than the wild-type strain, which may mean that loss of this specific chemotactic response leads to increased motility overall (Fig. 2I).

We competed strains missing motility genes with WT strains in two ways: first, we combined cultures in a 1:1 ratio at lag phase and incubated them through LTSP, so that the strains experienced all phases together (Fig. 2A, C, E, and G). Second, we competed for strains with each other only in LTSP. We did this by growing both WT and mutant strains on their own for 4 days, into the beginning of LTSP, and then combining the cultures in a 1:1 ratio and incubating for another 11 days (Fig. 2B, D, F, and H). The most surprising result was observed in competition between the Δ*cheA* strain and the wild-type strain (Fig. 2A and B). *cheA* encodes the histidine kinase protein of the two-component signaling pathway, CheA, which signals the flagellar motor switch to change directions as it senses chemoattractants and oxygen (25). When competitions began during lag phase, the Δ*cheA* mutant outcompeted the wild-type cells after 24 h of incubation by a 10,000-fold difference, with wild-type cells decreasing from their inoculated cell density (~10^7^ CFU/mL at inoculation to between 10^5^ and 10^6^ CFU/mL at 24 h) (Fig. 2A). A drastic decline in wild-type cell densities continued and by day 11 there was no detectable wild-type strain present. However, after normal cell growth for the Δ*cheA* mutant strain, the mutant strain maintains a cell density of ~10^8^ CFU/mL after 48 h of incubation (Fig. 2A). For competitions beginning in LTSP, we found that this effect was not as clear (Fig. 2B). In only one out of three competitions, the WT strain decreased in CFU/mL after 11 days of incubation, and in the other two competitions, mutant and WT strains were similar in CFU/mL throughout. It was possible that Δ*cheA* strains could grow so quickly when competing with WT cells that the WT cells could not recover, although the growth rates were similar to those when they were grown on their own (Fig. S1). Comparatively, there was little outgrowth of Δ*cheA* cells in competition only in LTSP.

*flgB* encodes a protein in the flagellar basal body, and unlike *cheA*, is not transcribed by sigma F (26). Cells missing *flgB* were not able to form flagella and, therefore, were nonmotile, as observed in our motility assays (Fig. 2I). After being inoculated at approximately the same cell density (10^7^ CFU/mL) as wild-type cells, the Δ*flgB* strain did not grow whereas wild-type cells did, which led to the wild-type strain being present in the culture at ~10- to 100-fold higher after 24 to 48 h of incubation (Fig. 2C). However, after 72 h of incubation, the wild-type cell counts decreased, and the wild-type and mutant cells had a similar cell density of between 10^6^ to 10^8^ CFU/mL for the rest of the incubation period (Fig. 2B). In competition during LTSP only, the Δ*flgB* strain was present at a similar cell density to the WT strain (Fig. 2D).

As mentioned above, *fliA* encodes sigma F, and so cells missing *fliA* should not be able to form flagella or perform chemotaxis (18), as confirmed in our motility assays (Fig. 2I). For competition between the Δ*fliA* strain and the wild-type strain starting in lag phase, both strains showed similar densities after 24-h of incubation (Fig. 2E). The *ΔfliA* mutant showed a sharp decrease after 48 to 72 h of ~100 to 1000-fold, whereas the wild-type strain only decreased by ~10-fold after 48 h and ~100-fold after 72 h. By day 6 of incubation, the wild-type and mutant cells both recovered to a cell density of ~10^8^ CFU/mL and remained at similar cell densities for the rest of the incubation period (Fig. 2E). Similarly, in competition during LTSP only, the Δ*fliA* strain was present at a similar cell density to the WT strain throughout (Fig. 2F).

*tar* encodes a methyl-accepting chemotaxis protein that senses the chemoattractant aspartate and other amino acids (25). Cells missing *tar* should still be able to move but may not be able to sense some of the amino acids that *tar* generally senses. There is some overlap with other methyl-accepting chemotaxis proteins (27). In our motility assays, strains missing the *tar* gene were more motile than WT cells (Fig. 2I). In the competition of the *Δtar* strain and wild-type cells starting in lag phase, both strains grew similarly throughout long-term incubation (Fig. 2G). In competition during LTSP only, in one competition the Δ*tar* strain was at a lower cell density by 11 days of incubation, whereas in the other two competitions the Δ*tar* and WT strains had similar cell densities throughout incubation (Fig. 2H). None of the mutant strains grew differently than wild-type strains when grown without competition from wild-type cells in monoculture (unpublished data).

Given these observations, in comparing growth phenotypes to motility phenotypes, these results suggest that our hypothesis that increased motility would allow cells to have an advantage in survival in LTSP was not supported because a gain of motility (from loss of *tar*) did not give cells an advantage, and the loss of motility (from loss of *cheA*, *fliA*, or *flgB*) did not give cells a disadvantage (Fig. 2).

### Mutation analysis of RNA-seq data identified mutations potentially involved in motility.

Because motility did not seem to affect the cells’ ability to survive in LTSP, we hypothesized that we could identify mutations in aged populations that were isolated to one gene that had pleiotropic effects, and the increase in motility was a “hitchhiker” to other effects which were advantageous to the cell. To identify mutations that we knew were associated with the increase in motility-related genes, we identified mutations in our RNA-seq data using the program *breseq* in population mode (28).

We identified 5 unique mutations in the two aged populations, and one mutation present in both populations (Table 2). Because we used the “population” setting in *breseq*, we were able to determine what percentage of the reads, and therefore likely the population, contained each mutation. Two of the unique mutations were both mapped to the *sspA* gene, encoding the stringent starvation protein (29), which had previously been noted as a gene that is frequently mutated in long-term cultures that often confer an advantage to cells (6, 7). One *sspA* mutation (R65C) was present in 66.9% of the reads in aged population A, and the other mutation (V25F) was present in 79.5% of the reads in aged population B. SspA regulates transcription through both regulation of H-NS (30) and directly via RNA polymerase (31), and R65 is an essential amino acid for its interactions with RNAP (31). Loss of function of SspA may be responsible for regulating motility via the DNA-binding regulatory protein H-NS. SspA negatively regulates H-NS, which in turn negatively regulates the expression of *csgD* (29). Loss of function of SspA may lead to the higher activity of H-NS, which would then lead to lower expression of *csgD*, as we saw in our transcriptome data (Table 1). CsgD is a negative regulator of *fliA* (18). Thus, in these cells, the repression of *csgD* might lead to a derepression of *fliA*, and, therefore, other motility- and chemotaxis-related genes. Further, H-NS is a positive regulator of *fliA* (along with other flagellar-related genes) (32), so higher activity of H-NS could also lead to a direct increase in *fliA* expression.

**TABLE 2:** Single nucleotide polymorphisms (SNPs) identified in aged populations via RNA-seq

Gene	SNP identified	% of population
Population A		
*hokB* and *mokB*	Y29C (TAC→TGC) and L48L (CTA→CTG)	68.6%
*argV*	noncoding (35/77 nt)	67.2%
*sspA*	R65C (CGT→TGT)	66.9%
*leuQ*	noncoding (48/87 nt)	55.5%
Population B		
*argV*	noncoding (35/77 nt)	65.3%
*sspA*	V25F (GTC→TTC)	79.5%
*cytR*	A257T (GCG→ACG)	85.9%

There was also a mutation mapped to *argV* in both populations, encoding an arginine tRNA (ACG) (33). Because this exact mutation was present in both aged populations, there was a chance it occurred during the initial overnight growth, or it could be due to molecular convergence leading to selection for this allele.

In population A, we also identified mutations that mapped to the *hokB* and *mokB* genes (68.6% of reads), which overlap in the genome. HokB is part of the HokB-SokB type 1 toxin-antitoxin system (34). MokB is a putative regulator of HokB (34). However, the base change which leads to a missense mutation in HokB leads to a silent mutation in MokB. We also identified a mutation in *leuQ* (55.5% of reads), another gene encoding a tRNA, this one for leucine (CAG) (35).

In population B, the only other mutation identified was mapped to *cytR* (85.9% of reads), a regulator of nucleoside metabolism (19). Like *sspA*, *cytR* frequently accumulates mutations in cells incubated to LTSP, and *sspA* and *cytR* mutations often occur in the same populations (6, 7).

## DISCUSSION

We identified multiple genes that were differentially expressed during log phase in populations of E. coli that have been incubated into LTSP compared to their unaged parental strain. Several genes involved in motility and chemotaxis had higher expression in cells that had been aged into LTSP (Table 1), leading to increased motility. The increase in motility-related genes or the motility phenotype occurred in multiple aged cultures, suggesting that the phenomenon of increased motility may be common in cells adapted to LTSP. Because the RNA-seq data showed overexpression in motility-related genes and aged populations were more motile, we hypothesized that increased motility would be advantageous in LTSP. We would not have hypothesized this *a priori*. Because these cultures were shaken in tubes, we originally thought cells would not need their motile or chemotactic abilities to access nutrients or oxygen. Contrary to our predictions, based on single-gene deletion mutations (Fig. 2) we determined that motility does not correlate with survival in LTSP.

However, we did observe a surprising result when *cheA* was deleted. These cells could outcompete wild-type cells beginning in log phase and throughout the E. coli life cycle (Fig. 2A). When CheA was inhibited, the cells performed little to no clockwise movement, which meant that the cells would mostly be engaging in counterclockwise movement and long smooth runs (25). Two hypotheses could explain this result. First, the long smooth runs could be giving an advantage to the mutant cells over the wild-type cells, which would be performing a normal run and tumble pattern. Long smooth runs would allow cells to steadily move toward chemoattractants. However, because the cells were grown and competed in test tubes in a shaking incubator, it seemed unlikely that the long runs would give an advantage in that environment because cells were being moved around the tubes likely without needing to use their flagella. A second hypothesis is that the *ΔcheA* mutant was conserving energy by not having to switch between the run and tumble movement. The conservation of energy could provide an advantage to the cells.

Because loss of motility did not lead to a loss of ability to survive in LTSP, we then hypothesized that the increase in motility may be a pleiotropic effect of a mutation in a regulatory gene. We thus identified multiple mutations in the aged populations using the RNA-seq data. Unique mutations in the gene *sspA* were identified in both aged populations (Table 2), which may lead to an increase in motility via increased activity of H-NS. Along with explaining the increase in motility-related genes, the increased H-NS activity could also explain the decreased expression of acid-resistance genes in aged populations (Table 1) because it is a negative regulator of many of those genes (36). We hypothesize that this loss or decrease in function of SspA leads to the expression differences and phenotypes we observed in the aged populations, and ongoing testing will determine how this change allows cells to survive in LTSP.

We also identified mutations in *cytR* (Table 2), which is a negative regulator of other genes that were more highly expressed in aged cells: *nupG*, *tsx*, *cdd*, and *udp* (Table 1) (19). The mutations in *cytR* and other identified mutations likely did not affect the motility phenotype we observed. However, these data indicate that identifying mutations in RNA-seq data can be useful in explaining expression changes in aged populations. Understanding which mutations occur in long-term cultures, and if and why they were advantageous, gives insight into how cells adapt to this stressful condition.

## MATERIALS AND METHODS

### Bacterial strains and growth conditions.

The E. coli K-12 lineage MG1655-derivative PFM2 (37) and a chloramphenicol resistant (PFM2-CM) derivative of PFM2 (7) were used in this study. To start overnight cultures of cells, frozen stocks of cells stored in a −80°C freezer in 5 mL of Difco Luria-Bertani Broth (LB; BD Difco) were inoculated and incubated overnight (16 to 18 h) at 37°C in a 150 mm × 18 mm test tube in a shaking incubator at 200 RPM. These frozen stocks consist of a population of cells originally started from a single clone. A 1:1000 dilution was then inoculated into fresh 5 mL of LB and incubated as described above. The long-term cultures were incubated under these conditions for 10 days with no addition of nutrients. After 10 days, they were stored in a 10% glycerol solution in LB at −80°C.

Mutant strains were created for competition assays against wild-type nonmutant (PFM2) cells using bacteriophage P1 transduction. Strains in the KEIO collection, which is a collection of single-gene knockout mutants with a kanamycin resistance cassette in the open reading frame coding regions (38), were used as donors to generate the desired P1 lysates to transfer mutant genotypes. P1 transduction protocol as described previously (15) was used.

### RNA preparation, sequencing, and analysis.

Cultures of wild-type and aged cells were started as described above, incubated overnight, and diluted at 1:1000 the next day. Two cultures of the wild-type strain and the aged strain were grown for 4 h into the log phase. RNAprotect reagent (Qiagen) was added per the manufacturer’s instructions. Cells were pelleted and frozen at −80°C until total cellular RNA extraction was performed using the RNeasy Protect Bacteria Minikit (Qiagen). Total RNA was processed, assessed for quality, and sequenced by the University of Southern California Genomics Core, where rRNA was removed using the Ribo-zero kit (Illumina, Inc.). Seventy-five base pair single-end reads were generated from each sample on the Illumina HiSeq 2500 platform. We received an average of 8.8 million reads per sample, with a range of ~7.5 million to 10.4 million reads per sample. Using a custom Python pipeline, we used TopHat2 to align the raw reads to the E. coli MG1655 genome (39), SAMtools to generate binary alignment files (BAM) (40), and HTSeq to calculate read counts per gene and per sample (41). In all samples, 96.5% of reads mapped to our reference genome (Escherichia coli K-12 MG1655). All rRNA gene reads and any gene that had no expression were removed from the data set. We used the HTSeq output files as input to analyze gene expression levels using DESeq2 (42), with comparisons between unaged and aged populations. We considered genes to be differentially expressed between these populations if the log_2_ fold change was greater than or equal to 2, and if the q-value was less than or equal to 0.05.

### Motility assays.

Motility assays were used to assess the movement differences between the samples. Swimming motility assays were performed using 0.18% semisolid agar (0.54g Bacto Agar [BD Difco™ Bacto™], 6.0g LB broth powder [BD Difco™], and 300 mL MilliQ water). The semi solid agar was autoclaved for 15 min at 121°C. 17.6 mL of the semisolid agar was poured into 100 mm × 15 mm plastic Petri plates creating 2.2 mm thick semisolid agar in each plate. Overnight cultures of the wild-type (unaged) cells along with overnight cultures of the aged or mutant populations were inoculated in liquid media as described above. The cells were then further diluted in a 1:1000 ratio in 5 mL of LB and placed in the 37°C shaking incubator for 4 h. After the incubation, the center of the semisolid agar plates was inoculated with cells from each culture using an inoculation loop without touching the bottom of the plate. The plates were then placed in a 37°C incubator for 12 h. The zone of motility was recorded by measuring the diameter of the halo or the distance the cells traveled from the center inoculation point. A Welch's or Student's *t* test was used to determine the significance of the difference between the aged and unaged populations and wild-type and mutant populations using the ggpubr package in R.

### Growth assays.

Competition assays were performed in liquid culture using 5 mL of LB broth. Competitions were completed in two ways: first, cells were competed starting in lag phase using cultures that were inoculated with a 1:1000 dilution of wild-type cells with a chloramphenicol-resistance marker (PFM2-CM) that were incubated as described above, and a 1:1000 dilution of mutant cells (*ΔfliA*, *ΔflgB*, *Δtar*, or *ΔcheA*) with a kanamycin-resistance marker that was incubated as described above. The cells were placed in the shaking incubator at 37°C and allowed to compete for resources for 11 days. Second, cells were competed in LTSP only by first incubating cells in monoculture for 4 days into LTSP, and then mixing them in a 1:1 ratio (2.5 mL each) into a new tube. Serial dilutions were performed to measure the cell densities of both strains on days 0, 1, 2, 3, 6, and 11 of incubation (6). The dilutions were plated on chloramphenicol LB agar plates and kanamycin LB agar plates to distinguish between the cell types, with wild-type cells growing on chloramphenicol plates and mutant cells growing on kanamycin plates.

Monocultures of the wild-type cells (PFM2) and the *ΔfliA*, *ΔflgB*, *Δtar*, and *ΔcheA* mutants were also incubated as described above, with each strain grown in its own culture. The cells were plated on LB agar plates with no antibiotic.

### Mutation analysis.

The RNA-seq Illumina reads were aligned to the E. coli K-12 (strain MG1655) genome (NCBI reference sequence NC_000913.3) using the population analysis settings with the BreSeq package in Python (28).

### Data availability.

Both count files derived from HTseq and raw sequence reads have been deposited in NCBI’s Gene Expression Omnibus (41) and are accessible through GEO Series accession number GSE188790.
