# Supplementary material for: Parallel Evolution towards Increased Motility in Long-Term Cultures of Escherichia coli, Even Though Motility was Not Required for Long-Term Survival
Source: Microbiol Spectr. 2022 Jun 23;10(4):e02330-21. doi: 10.1128/spectrum.02330-21 (PMC9431438; doi:10.1128/spectrum.02330-21)
Supplement: Supplemental file 1 — Supplemental material. Download spectrum.02330-21-s0001.pdf, PDF file, 0.2 MB [file spectrum.02330-21-s0001.pdf]

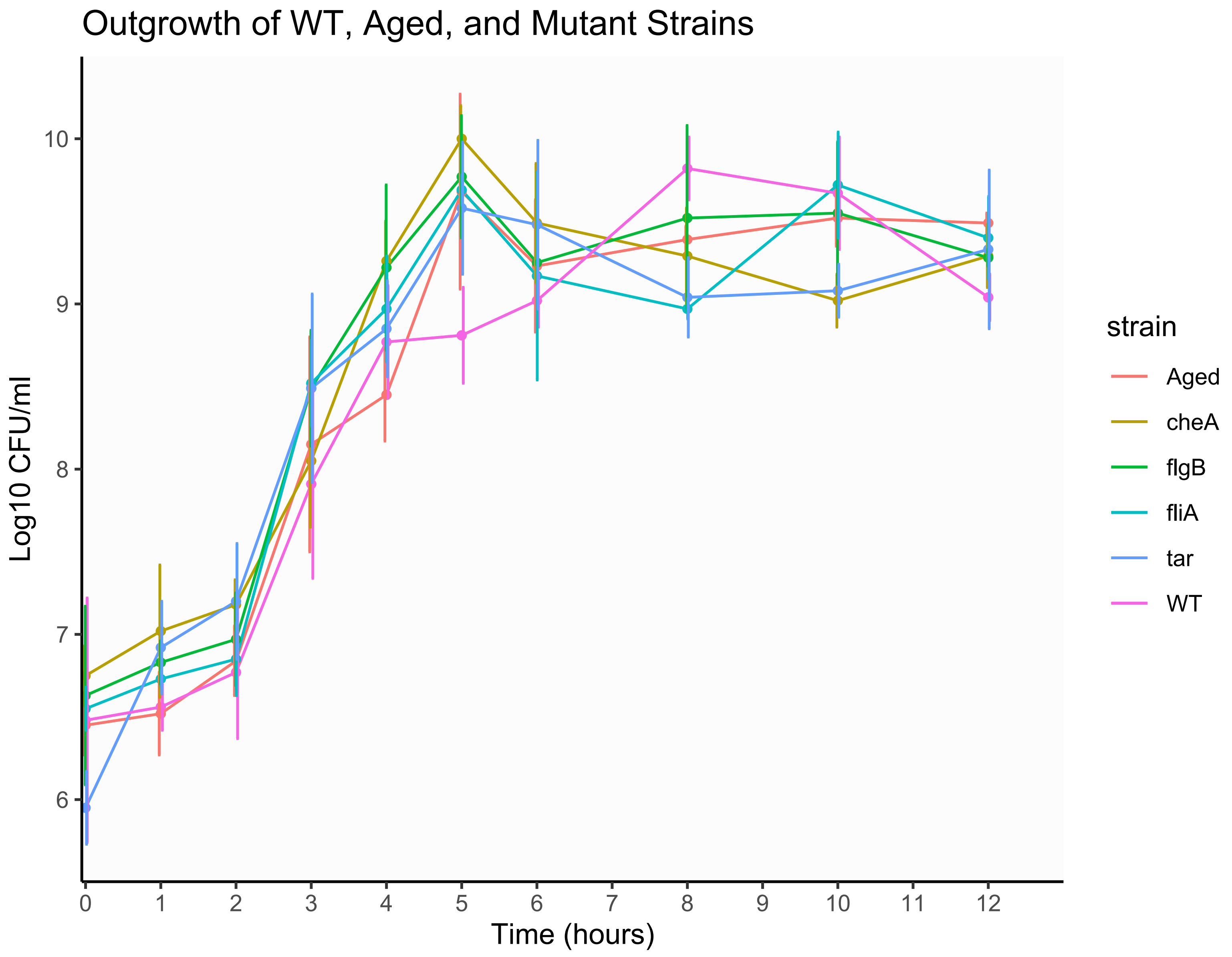

Figure S1. Wild type, aged, and mutant strains have similar growth in lag, log, and stationary phases. Cultures of wild type (WT), aged, and mutant strains used in this study were inoculated into LB and incubated for 12 hours. For eight hours, CFU/ml was measured every hour, and for the remaining time measured every two hours. Each line represents three replicate samples, and error bars represent standard deviation. Both growth phase and CFU/ml were similar amongst all strains throughout the growth period.
